# A Case of Diagnostic Difficulty: Transient Loss of Consciousness in Artery of Percheron Infarct

**DOI:** 10.7759/cureus.12918

**Published:** 2021-01-26

**Authors:** Aishwarya Sharma, Dinesh Bande, Abhishek Matta

**Affiliations:** 1 Infectious Diseases, Sanford Health, Fargo, USA; 2 Internal Medicine, University of North Dakota School of Medicine, Fargo, USA; 3 Internal Medicine, University of North Dakota, Fargo, USA

**Keywords:** loss of consciousness, artery of percheron, aop infarct

## Abstract

The artery of Percheron (AOP) is a rare anatomical variant of the paramedian thalamic vessels in 7-10% of the general population. An AOP infarct can present with rare clinical manifestations like transient loss of consciousness (LOC) and lethargy, as was seen in the patient whose case is discussed in this report, due to the plethora of regulatory inputs and outputs by the thalamus, which cannot be compensated for because of the absence of anastomotic connections. The AOP supplies the reticular activating system (RAS), which regulates consciousness. Ischemia to this area from an AOP infarct can result in the transient LOC, which our patient experienced. The AOP is a small vessel that is often missed on a CT angiogram (CTA) alone due to low resolution. As a result, it is imperative that clinicians utilize MRI to diagnose AOP infarcts in patients who present with symptoms that raise concerns for decreased bilateral thalamic function and transient LOC.

## Introduction

The medial portion of the thalamus and the rostral midbrain are supplied by the artery of Percheron (AOP) in 7-10% of the general population [1]. This blood vessel is a rare derivative of the paramedian thalamic vasculature [1]. Obstruction of the AOP can manifest as diverse clinical presentations as a result of the wide range of neurological functions controlled by the thalamus including behavior, cognition, and sensation. Patients with an AOP infarct account for 0.4-0.5% of all ischemic strokes and can present with altered mental status, transient or episodic loss of consciousness (LOC), and memory impairment [2-5]. In a retrospective study comparing detection rates for AOP strokes among different imaging modalities, MRI imaging was reported to be able to identify 100% of AOP cases in comparison to only 50% by CT scan [4]. Due to a low rate of suspicion for cerebrovascular accident (CVA) in this area as well as the difficulty in distinguishing occlusions in small arteries, AOP infarct cases are often not observed on an initial CT angiogram (CTA). The initial investigation for our patient was done at an outside ER. Since the presentation was syncope, they did not delve more into stroke workup beyond a CTA as they felt a cardiac cause was more probable. Delayed identification of the ischemia in conjunction with variable clinical presentations can delay the diagnosis and treatment, resulting in subsequent deficits. Increased importance should be placed on identifying AOP infarcts in patients presenting with generalized neurological deficits and lethargy since its occlusion can diminish bilateral function. In this report, we discuss the case of a patient with a rare presentation of an AOP infarct.

## Case presentation

A 79-year-old healthy male, with no known history of hypertension, diabetes mellitus, or stroke in the past, presented to a local emergency room with complaints of three syncopal episodes. As part of the syncope workup at the outside hospital, EKG showed no acute ST changes. Labs and vitals were unremarkable, as seen in Table 1. CTA of the head and neck did not show any vessel occlusion. Syncope was thought to be secondary to cardiovascular etiology, and hence he was discharged home on a Zio patch to monitor for cardiac arrhythmia and with instructions to attend an outpatient follow-up with primary care.

**Table 1:** Lab values at the outside hospital WBC: white blood cells; Hb: hemoglobin; BUN: blood urea nitrogen

Variables	Value	Reference range
WBC	7	4.0-11.0 K/uL
Hb	14.8	13.5-17.5 g/dL
Platelet count	102	140-400 K/uL
Sodium	139	135-145 meq/L
Potassium	4.8	3.5-5.3 meq/L
Chloride	106	98-106 meq/L
Bicarbonate	25	22-29 mmol/L
Creatinine	1.2	0.80-1.30 mg/dL
BUN	17	7-20 mg/dL
Troponin I	0.001	0.000-0.028 ng/mL

**Figure 1:**
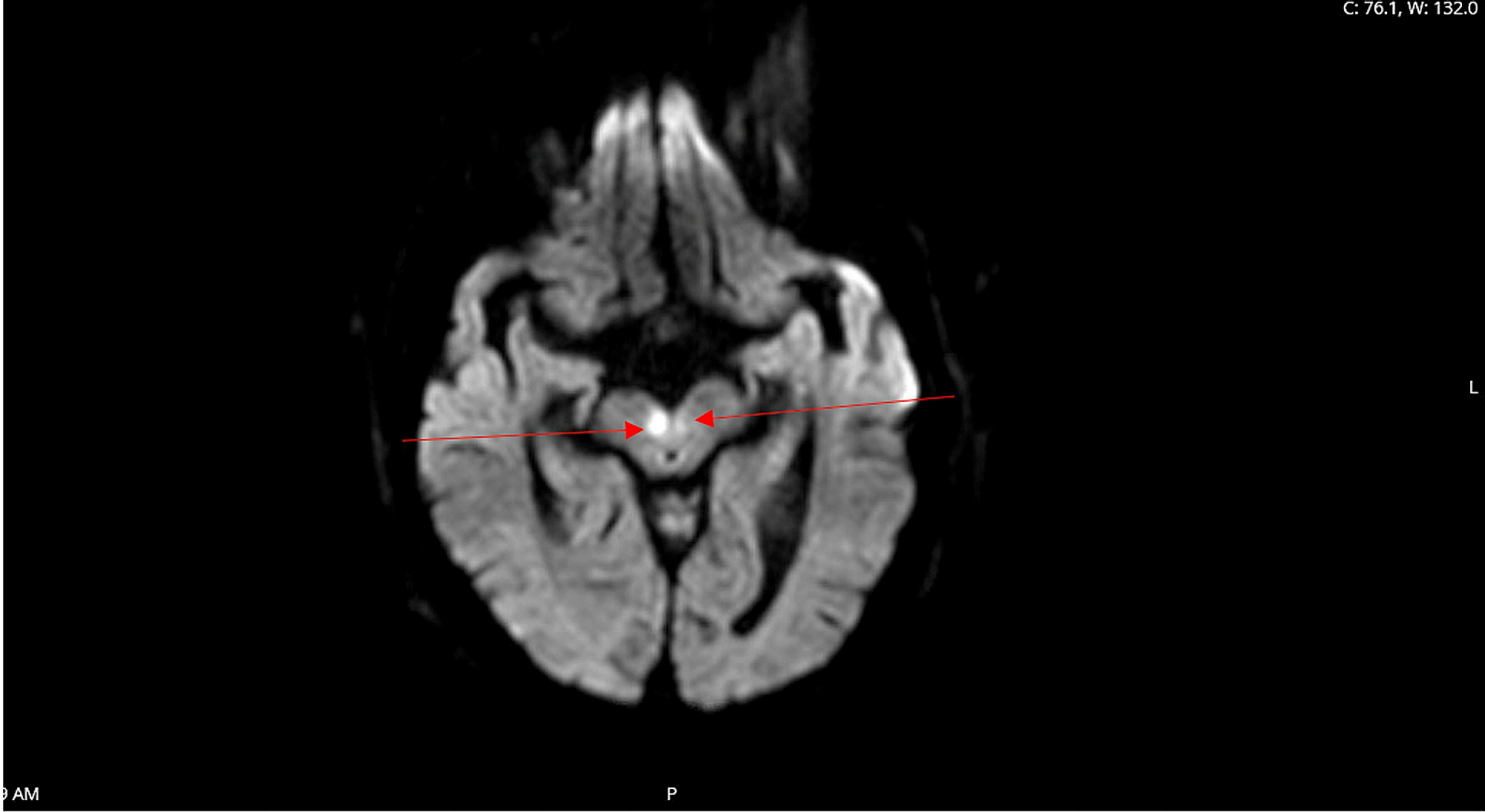
Bilateral thalamic CVA (red arrows) CVA: cerebrovascular accident

**Figure 2:**
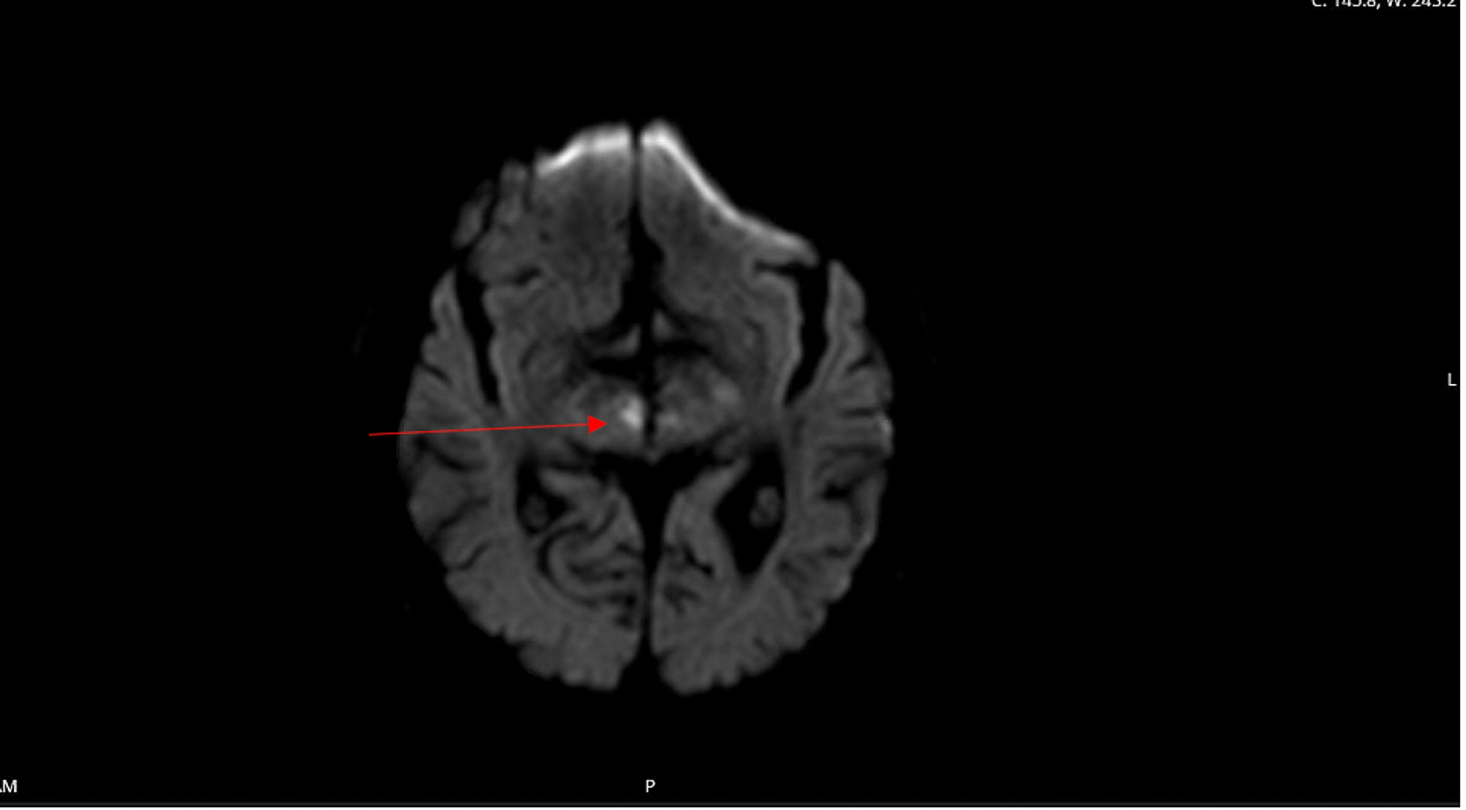
Right midbrain involvement suggestive of stroke from occlusion of AOP (red arrow) AOP: artery of Percheron

He presented to our emergency room two days later with diplopia, left-sided weakness, slurry speech, and lethargy. Physical examination revealed left hemiparesis, ophthalmoplegia, dysarthria, and decreased sensation over the left face, arm, and leg. The patient was arousable and was oriented to place, time, and people. His Glasgow Coma Scale (GCS) score was 13. CT scan of the head showed no acute process. CTA of the head and neck showed no large-vessel occlusion but showed severe stenosis of the origin of the superior division M2 segment of the right middle cerebral artery (MCA) and minimal-to-moderate stenosis of the origin of the inferior division M2 segment of the left MCA. It also showed diffuse bilateral intracranial atherosclerotic disease. MRI of the brain revealed bilateral thalamic CVA with right midbrain involvement, which was suggestive of stroke from occlusion of the AOP (Figure 1, Figure 2). The lab values in the emergency room are listed in Table 2.

**Table 2:** Lab values in the emergency room WBC: white blood cells; Hb: hemoglobin; INR: international normalized ratio; LDL: low-density lipoproteins; HbA1c: glycated hemoglobin; IgG: immunoglobulin G

Variables	Value	Reference range
WBC	7.9	4.0-11.0 K/uL
Hb	15	13.5-17.5 g/dL
Platelet count	78	140-400 K/uL
Glucose	104	70-100 mg/dL
Sodium	141	135-145 meq/L
Potassium	4.2	3.5-5.3 meq/L
Creatinine	1.17	0.80-1.30 mg/dL
Troponin I	0.017	0.000-0.028 ng/mL
INR	1.1	2.0-3.5
LDL	102	0-129 mg/dL
HbA1c	5.5	<5.7%
Cardiolipin Ab IgG	<14	<=14 GPL
Cardiolipin Ab IgM	65	<=12 MPL
Beta-2-GPI IgG	<9	<=20 SGU
Beta-2-GPI IgM	<9	<=20 SMU

Neurovascular service was consulted, and the patient did not receive any tissue plasminogen activator (tPA) given that he was outside of the window. Given the lethargy, the patient was admitted to the neurocritical ICU for close monitoring. He was started on aspirin and atorvastatin. The GCS score improved to 15 in a few hours and the patient did not require any intubation. Transthoracic echocardiogram (TTE) showed an ejection fraction of 65% and mildly dilated left atrium with no evidence of a shunt. Neurological function was back to normal within 48 hours of the admission. There were no focal deficits at the time of discharge. Given his stroke and no clear etiology, a loop recorder was placed for long-term monitoring of atrial fibrillation. His mental status and left hemiparesis improved, and the patient was discharged home with outpatient physical therapy and occupational therapy. However, diplopia did not improve during the hospital stay. An eye patch was provided for his diplopia.

The patient still had diplopia at the one-month follow-up in the clinic although motor function had improved. Diplopia was corrected with prisms glasses by the six-month clinic follow-up. The follow-up on loop recorder at one year did not reveal any atrial fibrillation.

## Discussion

An insult to the thalamus due to occlusion of the AOP may result in variable neurological symptoms since the thalamus is one of the main feedback structures in the body. It comprises several nuclei that help to regulate sensation, behavior, movement, cognition, and emotion, and hence ischemia in this area impairs function throughout the body and makes diagnosis more difficult. In addition to the complexity in function, the thalamus is supplied to different degrees by the polar artery, the inferolateral artery, the paramedian artery, and the posterior choroidal arteries [1]. Since this vascular supply lacks functional anastomosis with other vasculature in the region, it increases the damage that can result due to ischemia in this area. The AOP is present in only one-third of human brains [2]. It originates from the paramedian arteries, which receive input from the amygdala, limbic basal ganglia, and olfactory cortex [3]. Patients presenting with an insult to this area display a temporal LOC, vertical gaze palsy, and impaired cognition in addition to cardinal stroke symptoms such as aphasia, dysarthria, and oculomotor disturbance [4-5].

Visceral responses are regulated by the zona incerta, which is located in the center of the diencephalon [6]. Lowered blood pressure and lack of consciousness arise following the activation of the zona incerta and the ventrolateral diencephalon due to the blockage of sympathetic efferent input from the posterior thalamus, which receives an afferent from the anterior insular cortex [7,8]. Typically, the zona incerta is supplied by the left and right PCA since it is part of the thalamic nuclei; however, in some individuals, it can be supplied by an uncommon anatomic variant-AOP, which lacks anastomotic connections [9]. The reticular activating system (RAS) is a network of neurons located in the brain stem with projections to the hypothalamus anteriorly and thalamus posteriorly. The RAS is responsible for mediating consciousness by providing communication between the brainstem, thalamus, and cerebral cortex. Ischemia to the RAS due to an AOP infarct can lead to LOC [10].

Our patient initially presented with syncope. Syncope, by itself, has a very broad differential diagnosis, CVA being one among them. Since our patient did not have any focal symptoms at the time, the syncope was thought to be secondary to cardiovascular etiology and further investigations were performed in that direction. However, the patient’s clinical condition worsened with the emergence of focal motor difficulties and worsening mental status, which led to the diagnosis of CVA. The delay in the emergency of classic symptoms of CVA created a diagnostic dilemma and led to a delay in diagnosis. Fluctuating level of consciousness has been noted in patients with acute CVA [11]. We should be aware that acute CVA patients can present with fluctuating mental status, and hence a high degree of suspicion and appropriate imaging techniques are necessary to diagnose acute CVA when patients present without focal symptoms. Also, this case emphasizes the need to provide close monitoring and follow-up in patients who are discharged from the ER after syncope evaluation to keep track of recurrent syncope and the emergence of new symptoms that can indicate the etiology of the syncope.

## Conclusions

An AOP infarct can have variable presentations due to the wide range of functions controlled by the thalamus and heterogeneity in thalamic vascular anatomy. This case highlights the need for physicians to consider AOP stroke presenting as thalamic pathology in patients with generalized neurological symptoms and fluctuation of mental status without the cardinal signs of stroke so that prompt intervention can be initiated to minimize deficits in patients. Syncope patients who are managed and discharged from the ER should be closely monitored in the immediate post-discharge period for the emergence of new symptoms that may indicate the etiology of the syncope.

## References

[1] Chen XY, Wang Q, Wang X, Wong KS (2017). Clinical features of thalamic stroke. Curr Treat Options Neurol.

[2] Caruso P, Manganotti P, Moretti R (2016). Complex neurological symptoms in bilateral thalamic stroke due to Percheron artery occlusion. Vasc Health Risk Manag.

[3] Blumenfeld H (2010). Neuroanatomy Through Clinical Cases. Neuroanatomy Through Clinical Cases. Sunderland: Sinaur Associates Inc.; 2010. Neuroanatomy Through Clinical Cases; pp.

[4] Xu Z, Sun L, Duan Y, Zhang J, Zhang M, Cai X (2017). Assessment of Percheron infarction in images and clinical findings. J Neurol Sci.

[5] Zappella N, Merceron S, Nifle C (2014). Artery of Percheron infarction as an unusual cause of coma: three cases and literature review. Neurocrit Care.

[6] Peruzzotti-Jametti L, Bacigaluppi M, Giacalone G, Strambo D, Comi G, Sessa M (2011). Life-threatening bradycardia after bilateral paramedian thalamic and midbrain infarction. J Neurol.

[7] Pitts-Tucker T, Small J (2018). Artery of Percheron: an unusual stroke presentation. BMJ Case Rep.

[8] Thurtell MJ, Halmagyi GM (2008). Complete ophthalmoplegia: an unusual sign of bilateral paramedian midbrain-thalamic infarction. Stroke.

[9] Qian J, Wu C, Peng J, Liu H (2017). Bilateral paramedian thalamic and midbrain infarction due to occlusion of the artery of Percheron in an elderly male: a case report. Neurol Sci.

[10] Kocaeli H, Yilmazlar S, Kuytu T, Korfali E (2013). The artery of Percheron revisited: a cadaveric anatomical study. Acta Neurochir (Wien).

[11] Li J, Wang D, Tao W, Dong W, Zhang J, Yang J, Liu M (2016). Early consciousness disorder in acute ischemic stroke: incidence, risk factors and outcome. BMC Neurol.

